# Bidirectional communication of the gut-brain axis: new findings in Parkinson’s disease and inflammatory bowel disease

**DOI:** 10.3389/fneur.2024.1407241

**Published:** 2024-05-24

**Authors:** Zhang Wanyi, Yan Jiao, Huang Wen, Xu Bin, Wang Xuefei, Jiang Lan, Zhou Liuyin

**Affiliations:** ^1^Department of Neurology, Chongqing Emergency Medical Center, Chongging University Central Hospital, Chongqing, China; ^2^Department of Nursing, Chongqing Emergency Medical Center, Chongging University Central Hospital, Chongqing, China; ^3^Outpatient Department, Chongqing Emergency Medical Center, Chongging University Central Hospital, Chongqing, China; ^4^Department of Respiratory Medicine, Chongqing Emergency Medical Center, Chongging University Central Hospital, Chongqing, China

**Keywords:** inflammatory bowel disease, Parkinson’s disease, gut-brain axis, short-chain fatty acids, gut microbiota

## Abstract

Parkinson’s disease (PD) and inflammatory bowel disease (IBD) are the two chronic inflammatory diseases that are increasingly affecting millions of people worldwide, posing a major challenge to public health. PD and IBD show similarities in epidemiology, genetics, immune response, and gut microbiota. Here, we review the pathophysiology of these two diseases, including genetic factors, immune system imbalance, changes in gut microbial composition, and the effects of microbial metabolites (especially short-chain fatty acids). We elaborate on the gut–brain axis, focusing on role of gut microbiota in the pathogenesis of PD and IBD. In addition, we discuss several therapeutic strategies, including drug therapy, fecal microbiota transplantation, and probiotic supplementation, and their potential benefits in regulating intestinal microecology and relieving disease symptoms. Our analysis will provide a new understanding and scientific basis for the development of more effective therapeutic strategies for these diseases.

## Graphical abstract

PD and IBD mechanism of gut microbes.
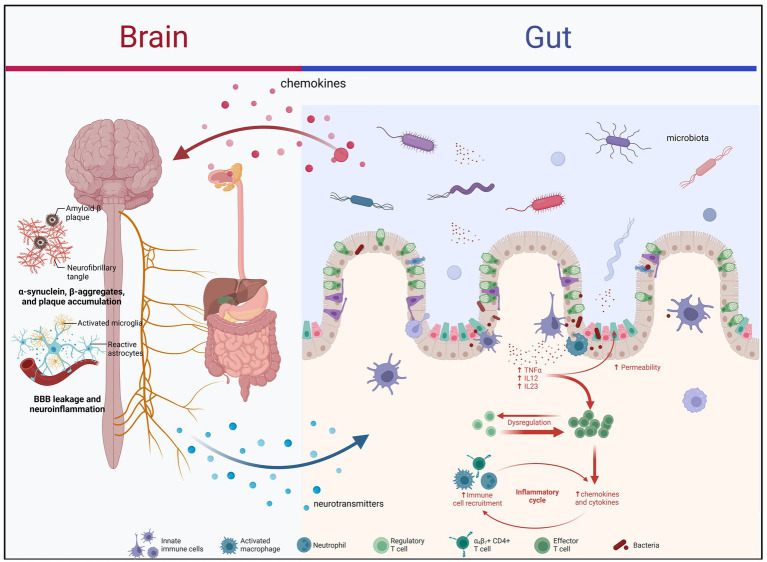

## 1 Introduction

Parkinson’s disease (PD) and inflammatory bowel disease (IBD) are increasingly affecting the global population. PD is a neurodegenerative disease characterized by bradykinesia, resting tremor, and myotonia; its pathogenesis involves various factors, including genetics, environmental factors, and age (1, 2). IBD, including Crohn’s disease and ulcerative colitis, is a group of diseases characterized by chronic intestinal inflammation, leading to abdominal pain, diarrhea, and hematochezia. Its etiology is complex and diverse, involving immune system abnormalities, genetic susceptibility, and intestinal microbial imbalance (3–5). The occurrence of PD and IBD may be intricately linked to each other, and the interplay between the gut and the central nervous system—the “gut–brain axis”—provides a potential biological basis for this link (6–8). Meanwhile, some studies have shown that IBD is a candidate diagnostic marker for PD (9), and the incidence of PD is significantly increased in IBD patients (10). Here, we will review the risk factors, pathophysiological mechanisms, and treatment methods of PD and IBD, with special focus on the role of gut microbiota and short-chain fatty acids (SCFAs). We aim to provide comprehensive information on the relationship between these two seemingly different but closely related diseases for future research on diagnostic and treatment strategies.

## 2 Epidemiology of PD and IBD2.1 epidemiological relationship between PD and IBD

Parkinson’s disease is the second most common neurodegenerative disease worldwide, and its prevalence is gradually increasing with the aging of the global population. PD mainly affects adults over 50 years of age; however, cases of PD have also been reported in younger age groups. Males are slightly more likely to develop PD than females, and most cases are thought to be sporadic, although familial clusters have been reported in about 10% of patients (11, 12). The increase in the prevalence and incidence of IBD in industrialized and high-income countries can be attributed to changes in environmental factors, lifestyle, and genetic susceptibility. Although IBD can occur at any age, it is most common in adolescents and young adults, and its incidence is similar between men and women. However, family history is an important risk factor for IBD, and individuals with family history have a significantly increased risk of developing the disease (13–15).

### 2.1 Risk of PD in patients with IBD

A meta-analysis of nine studies involving 12,177,520 patients revealed that the incidence of PD in patients with IBD was higher than that in the general population (RR = 1.24; *p* < 0.001) (10). Further, the results of another meta-analysis confirmed that patients with IBD had a higher incidence of PD compared with the general population (OR = 1.30; *p* = 0.024) (16). However, it is unclear whether the risk of IBD in patients with PD is different from that in the general population. The findings of existing studies are inconsistent, and sufficient epidemiological studies are not available. A cohort study involving 1968 patients with PD and 6,792 controls was conducted in Taiwan in 2015. The authors found that the prevalence of IBD between the two groups was not significantly different (*p* = 0.561) (17). A Swedish case–control study involving 39,652 patients with IBD and 396,520 controls revealed that patients with IBD were more likely to have PD at the time of IBD diagnosis compared with the control population (OR = 1.4; 95% CI: 1.2–1.8). Similar results were observed in the subgroup analysis of Crohn’s disease (OR = 1.6; 95% CI: 1.1–2.3) and ulcerative colitis (OR = 1.4; 95% CI: 1.1–1.9) (18). The 2019 update of the World Movement Disorder Society diagnostic criteria for the prodromal stage of PD specifies that IBD is a candidate diagnostic marker for PD. This implies reliable and credible evidence; however, the corresponding prospective studies are lacking (9).

### 2.2 Risk of IBD in patients with PD

Freuer et al. analyzed 463,372 IBD-related datasets (7,045 cases and 456,327 controls) and 1,474,097 PD-related datasets (56,306 cases and 1,417,791 controls). Mendelian randomization (MR) analysis using inverse variance weighting (IVW) showed that IBD was not associated with the risk of PD (OR = 0.98, *p* = 0.48) (19). MR analysis was performed using the data obtained from 59,957 patients with IBD (25,042 cases and 34,915 controls) and 1,474,097 patients with PD-related using five statistical methods, including IVW and robust adjusted profile score (RAPS). The risk of IBD in patients with PD was higher than that in the control population (IVW and RAPS OR values were 1.062 and 1.063, respectively; both *p* < 0.05). The results of the remaining three statistical methods were negative (19). Two-way MR was used to analyze 214,053 patients with IBD (3,753 cases and 210,300 controls) and 482,730 patients with PD (33,674 cases and 449,056 controls). The authors observed the incidence of IBD in PD (OR = 1.014; 95% CI: 0.967–1.063; *p* = 0.573) and that of PD in IBD (OR = 0.978; 95% CI: 0.910–1.052; *p* = 0.549) (20). Currently, there is a paucity of definitive evidence to establish a direct association between PD and IBD; however, epidemiological correlations between the two disorders have been observed. Additional prospective and mechanistic studies are needed to clarify the possible causal relationship between these two diseases and the specific biological pathways involved in their interaction. This information may provide important clues for the development of new strategies for the prevention and treatment of both diseases.

## 3 Pathophysiology of PD and IBD

### 3.1 Genetic factors

Several authors have reported common genetic variants associated with the risk of PD and IBD (21). The *NOD2/CARD15* gene may be a common risk gene for PD and IBD (22). This gene is located on chromosome 16 and encodes the NOD2 protein (23). Four single nucleotide polymorphisms (SNPs; *R702W*, *G908R*, *L1007fs*, and *P268S*) of the *NOD2/CARD15* gene are highly expressed in patients with Crohn’s disease and PD (22, 24, 25). However, Appenzeller et al. suggested that the three SNPs (*R702W*, *G908R,* and *L1007fs*) are not associated with PD (26). The NOD2 protein encoded by this gene plays an important role in maintaining intestinal homeostasis. Any mutation in the *NOD2* gene may increase the susceptibility to IBD in the corresponding population through nuclear factor-κb activation and cytokine response (27). The leucine-rich repeat kinase 2 (*LRRK2*) gene is located on chromosome 12 and encodes the LRRK2 protein (28). The *LRRK2* gene is one of the important pathogenic genes in PD, and it is also related to IBD (29). The *LRRK2* gene is highly expressed in peripheral blood mononuclear cells and may be involved in the inflammatory process. The expression of the *LRRK2* gene in B cells, T cells, and CD16+ monocytes was higher in patients with PD than that in healthy controls. The interferon-γ stimulation can increase the expression of this gene in the immune cells of patients with Crohn’s disease (30, 31). Several SNP sites in the *LRRK2* gene, including *N1437H*, *R1441C/G/H*, *Y1699C*, *I2*012T, *G2019S*, and *I2020T*, are the pathogenic mutation sites in PD, and *M2397T* is a risk site in Crohn’s disease. *N2081D* is the common risk locus of PD and Crohn’s disease, whereas *N551K* and *R1398H* are the common protective loci of PD and Crohn’s disease (29). *N2081D* is located in the kinase domain of the *LRRK2* gene and is associated with increased kinase activity of the *LRRK2* protein. *R1398H* is located in the Roc (Ras/GTPase-protein complex) domain of the *LRRK2* gene, and mutations in this site can inactivate the LRRK2 protein by increasing GTPase activity. *N551K* is not in any domain of the *LRRK2* gene, but *N551K* and *R1398H* show linkage disequilibrium (32, 33). Chronic neuroinflammation and intestinal inflammation are important pathophysiological processes in PD and IBD, respectively. Therefore, *LRRK2* gene mutations may mediate inflammatory responses by affecting the kinase and GTPase activities of the LRRK2 protein, thereby participating in the pathogenesis of these diseases (29). In addition to the *LRRK2* and *NOD2* genes, other genes associated with autophagy, such as *ATG16L1* (34, 35) and *IRGM* (36), are also associated with the pathology of IBD and may be involved in the pathogenesis of PD. These findings support the idea of abnormal autophagy as a shared pathophysiological feature of PD and IBD. Although these genetic findings provide valuable insights, the exact genetic link between PD and IBD remains a complex issue that requires further investigation. A better understanding of the genetic basis of these disorders may facilitate the development of therapeutic strategies targeting shared mechanisms, thereby providing patients with better treatment options and outcomes.

### 3.2 Immunomodulatory mechanisms

Chronic non-specific inflammation is often accompanied by structural and functional disorders of the gastrointestinal mucosal barrier. Crohn’s disease can affect any layers of mucosa from mouth to anus, whereas ulcerative colitis usually affects the lining of the colonic epithelium (37). C-reactive protein in the blood of patients with IBD is a reliable biomarker reflecting the severity of the disease. Atreya and Neurath observed an increase in the levels of tumor necrosis factor (TNF)-α and other cytokines in the gastrointestinal tract (38) and those of inflammation-related proteins, such as calprotectin, calgranulin C (also known as S100A12), and lactoferrin, in the feces of patients with IBD (39). Neuroinflammation is one of the important pathophysiological features of PD (40), and typical inflammation occurs in the gastrointestinal tract of patients with PD. The mRNA levels of TNF-α, interferons, interleukin (IL)-6, and IL-1β increase in the colon tissue of patients with PD. In addition, the levels of IL-1β, C-reactive protein, and calprotectin increase in the feces of these patients (41). α-Synuclein, an unfolded protein composed of 140 amino acid residues, is widely expressed in the human brain, especially in the synaptic terminals of neurons (42). This protein abnormally aggregates and forms fibrous structures called Lewy bodies in the brains of patients with PD; these structures are one of the most prominent pathological hallmarks of PD (43). Intestinal inflammation may cause brain inflammation through secondary systemic inflammatory response and eventually promote the abnormal accumulation of α-synuclein in the brain to induce PD (44). Immunohistochemical analysis of colon tissues from 8 patients with IBD (4 cases of Crohn’s disease and 4 cases of ulcerative colitis) and 4 controls showed that the level of α-synuclein in the non-inflammatory area of Crohn’s disease was 2.07 times higher that of the control group, and the level of α-synuclein in the inflammatory area was 2.35 times higher that of the control group (45). Kishimoto et al. fed drinking water containing 0.5% dextran sodium sulfate to A53T gene-mutant mice for inducing colitis. The results showed that the experimental group had earlier movement disorders, abnormal accumulation of α-synuclein and degeneration of dopaminergic neurons compared with the control mice (46). Abnormal accumulation of Lewy bodies in the enteric nervous system (ENS) has been detected in the early stage of PD, and ENS dysfunction may promote the development of gastrointestinal symptoms in PD patients (47). The ENS is the origin and entrance of pathological changes in PD, and spreads to the central nervous system through vagus nerve transmission, leading to further substantia nigra lesions. Enteric glial cells (EGCs), as the most abundant cells of ENS, are closely related to the intestinal microbiota (48) and respond to microbial invasion. Related studies have found that bacterial lipopolysaccharide (LPS) and IL-6 can activate EGC by binding to PRR on the membrane of EGC cells, trigger TLR4/NF-κB and other proinflammatory signaling pathways and the formation of NLRP3 inflammasome, and promote intestinal immune inflammation to clear pathogens (49, 50). EGC can exert immunosuppressive and anti-inflammatory effects by releasing GDNF and BDNF. After the release of GDNF, it can bind to RET on type 3 lymphocytes (ILC3), thereby activating ILC3 and promoting the release of anti-inflammatory factor IL-22 and the expression of repair genes in intestinal epithelial cells, thus protecting the inflammatory epithelium of colon (51). The released BDNF reduced the expression of nitric oxide synthase and pro-inflammatory factor IL-6 induced by LPS in mice by down-regulating the TLR4 receptor on EGC, and alleviated intestinal inflammation (52). Drokhlyansky et al. (53) applied single-cell sequencing to the analysis of human and mouse ENS and found that genes expressed in the intermuscular and mucosal EGCs were significantly different, and found that several PD risk genes were enriched in the ENS, among which NRXN1 and ANK2 were enriched in the EGC, indicating that the dysfunction of EGC may aggravate CNS disease. EGC reactive hyperplasia and its specific glial markers are found in the colon tissues of PD patients, and they appear in the early stage of PD (54). Therefore, EGC obtained by gastrointestinal mucosal biopsy and analyzed may be superior to α-syn in predicting early PD. In the latest study by Perez-Pardo et al. (55) immunofluorescence staining of fixed sections of the colon of dead mice also found that the expression of EGC-derived glial fibrillary acidic protein (GFAP) and α-syn was increased simultaneously, suggesting that EGC may also play a role in the pathological formation of intestinal α-syn. It has also been shown that α-syn can ascend to the central nervous system via the enteric glia Cx43 hemicchannel (through which glial-glial syncytial cells are connected as a pathway for intercellular communication between the gastrointestinal tract and the central nervous system) or the vagus nerve (56). It was further found that EGC also plays a role in the ascent of α-syn to the central nervous system. EGC in PD may be pathological activated, which may promote α-syn misfolding in ENS by participating in intestinal immune inflammation and help α-syn spread to the brain. In turn, α-syn may also act as an effector molecule to further promote the pathological activation of EGC. Therefore, chronic inflammation links these two diseases, providing potential targets for future therapeutic strategies. Future studies should focus on the specific mechanisms linking IBD and PD, especially the role of α-synuclein. In addition, strategies to intervene in intestinal inflammation should be explored to reduce the risk of PD or delay its progression.

### 3.3 Brain–gut axis: gut microbes and SCFAs

The “gut**–**brain axis” theory is based on experimental evidence indicating the link between the gut environment and the central nervous system. The theory proposes a connection between the emotional and cognitive centers of the brain with peripheral gut functions (57). The disruption of gut microbiota is closely related to autism, neurodegenerative diseases and emotional disorders (stress, depression, anxiety) (58). The composition of gut microbiota is affected by diet and environment, and the use of antibiotics is one of the important reasons for destroying the stability of gut microbiota (59). A rodent study showed that low-dose penicillin administered late during pregnancy and early after birth had long-term effects on mouse offspring, including altered gut microbiome composition, increased cytokine expression in the frontal cortex, altered blood–brain barrier integrity, and behavioral measures, with the mice showing anxitty-like behavior (60). Through metagenomic sequencing, Yang et al. found that a variety of phages and bacteria in the gut of patients with major depression were changed, among which the reduced abundance of Blautia and Eubacterium was significantly associated with depressive symptoms (61). In addition, a meta-analysis showed that Bacteroides, Paranobacillus, and Barnesiella were enriched in patients with depression, while Firmicutes, Spirospiraceae (UCG 003, UCG 002), and Bacteroides vulgaris were significantly depleted (62). More important, Kelly et al. (63) found that transplantation of “depressive microbiota” into germ-free mice induced depressive-like behaviors and features, including anhedonia and states of hopelessness. Therefore, by understanding the bidirectional communication system of the gut-brain axis, we can gain deeper insight into how changes in the gut microbiota affect brain function, which in turn affects individual emotional and behavioral performance. According to this theory, PD may be a consequence of intestinal dysbiosis or intestinal barrier dysfunction or both, which is caused by an unknown pathogen in the gastrointestinal tract. The main pathological manifestation of PD is Lewy body (LB), which is caused by the misfolding and aggregation of α-synuclein (α-syn) (64). Many studies have verified the “gut-brain axis” hypothesis that α-syn can spread from the gastrointestinal tract to the brain through the vagus nerve (65, 66). Kim and colleagues found that α-syn injected in the duodenum and pylorus of mice migrated through the vagus nerve to the substantia nigra, locus locus locus, olfactory bulb, cerebellum, and other brain regions to accumulate and precipitate, causing PD-related motor disorders and non-motor symptoms (65). A recent study (67) found that α-syn can promote the transmission of each other from the gut to the brain by interacting with Tau protein, triggering the loss of substantia nigra dopaminergic neurons. Furthermore, some researchers have used glucose probes to study the intestinal permeability of PD patients and found that the intestinal epithelial barrier in PD patients has similar dysfunction as that in patients with enteritis (68). A study using baboons as a model, published in Brain, found that α-syn not only travels from the gut to the brain, but also travels backwards (69). In recent years, studies have continuously revealed that psychological factors play an important role in the course of organic diseases (such as IBD) through the role of brain-gut axis (70). Psychological factors aggravate IBD by increasing intestinal permeability, changing intestinal flora and enhancing immune response mediated by brain-gut axis (71). Intestinal inflammation can cause psychological diseases. In recent years, animal studies have found that intestinal inflammation in mice with colitis can lead to increased serum C-reactive protein and cortisol levels, and lead to inflammation represented by increased cyclooxygenase-2 levels in the limbic system of the brain through the hypothalamic–pituitary–adrenal axis (HPA). Heightened reactivity and decreased brain-derived neurotrophic factor (BDNF), which is thought to be directly linked to psychological disorders such as anxiety (72). Therefore, pathological α-syn caused by intestinal barrier dysfunction moves from the intestine to the brain and induces PD, and PD patients are often accompanied by different degrees of gastrointestinal symptoms, which needs more basic and clinical research evidence to confirm.

PD and IBD are characterized by intestinal microbial dysbiosis (73, 74). The proportion of pro-inflammatory bacteria, such as Proteobacteria, increases, whereas the abundance of some beneficial bacteria (SCFA producers) decreases in the gut of patients with PD. This imbalance of microbiota may lead to impaired intestinal barrier function and increased intestinal permeability, allowing more pathogens and inflammatory molecules to enter the blood circulation. Ultimately, this affects brain function, promotes neuroinflammation, and induces abnormal aggregation of α-synuclein in PD (73). A decline in the production of SCFAs by bacteria, such as *Faecalibacterium* and *Roseburia*, in patients with IBD, which are essential for maintaining the health of the intestinal mucosa and suppressing inflammatory responses (75). The abundance of pro-inflammatory bacteria of the Enterobacteriaceae family is increased in patients with PD and IBD. The abundance of SCFA-producing bacteria, such as Prevotellaceae (Bacteroidota), Lachnospiraceae (including *Roseburia*; Firmicutes), and *Faecalibacterium* is decreased in these patients. While Verrucomicrobia, Verrucomicrobiaceae, the abundance of anti-inflammatory bacteria, including *Akkermansia*, Lactobacillaceae, and Actinobacteria (including *Bifidobacterium*) was heterogeneous. Anti-inflammatory bacteria, such as *Akkermansia*, Lactobacillaceae, and *Bifidobacterium*, can grow in an inflammatory environment, and their abundance increases later than the “intestinal inflammation” process of PD and IBD (76–78). Lactobacillus and Bifidobacterium can modulate the host’s immune response, enhancing gut health by improving mucosal barrier function and reducing inflammation. This is partly achieved through the production of short-chain fatty acids (SCFAs) like acetate, propionate, and butyrate, which have anti-inflammatory properties. These bacteria contribute to the strengthening of the gut barrier, preventing the translocation of harmful bacteria and endotoxins into the host’s circulatory system. This barrier function is crucial for preventing infections and maintaining immune homeostasis (79, 80).

Metabolites of gut microbiota, such as SCFAs, show similar changes in patients with PD and IBD. SCFAs are a group of saturated fatty acids with carbon atom number ≤ 6, including acetic acid, propionic acid, butyric acid, isobutyric acid, valeric acid, isovaleric acid, caproic acid, and isocaproic acid. They are mainly produced by intestinal microorganisms in the colon by fermentation of the dietary fiber (81). Mechanistic studies in animal models have shown that butyrate has beneficial effects in maintaining the integrity of the gastrointestinal mucosal barrier, quenching oxygen at the epithelial interface and acting as an immunomodulatory agent. Propionate has been reported to induce satiation by regulating the production of anorexigenic hormones and intestinal gluconeogenesis, while also affecting glucose metabolism. Butyrate has been suggested to be associated with anti-cancer and anti-inflammatory effects, but direct evidence for this is lacking (82). In addition, it has been found that the use of butyrate in animal models of Parkinson’s disease can improve dyskinesia and dopamine deficiency (83), while propionate seems to be negatively correlated with the Unified Parkinson’s Disease Rating Scale III (84). Shin et al. detected SCFAs in PD patients and found that the concentrations of acetic acid, propionic acid and butyric acid in feces decreased, while the concentrations of acetic acid and propionic acid in plasma increased in PD patients. The severity of the disease was negatively correlated with the concentrations of SCFAs in feces (except propionic acid), and positively correlated with the concentrations of acetic acid, propionic acid and valeric acid in plasma (84). SCFAs are involved in the occurrence of PD by affecting the integrity of the blood–brain barrier, the function of microglia, neuronal autophagy and apoptosis, the integrity of the intestinal barrier, and intestinal inflammation (85). SCFAs play an immunomodulatory role in IBD by participating in regulating the differentiation of innate and adaptive immune cells and the function of related cells (86). Chen et al. reported that SCFA concentration decreases in the feces of patients with PD, whereas it increases in blood, urine, and saliva. This phenomenon may be related to the effect of SCFAs on intestinal mucosal permeability (87). A meta-analysis of 12 studies involving 572 patients with IBD and 282 healthy controls showed that fecal concentrations of acetic acid, propionic acid, butyric acid, and valeric acid decreased in patients with IBD. However, subgroup analysis showed that the changes in fecal SCFAs in patients with Crohn’s disease were different from those in patients with ulcerative colitis. Acetic acid, valeric acid, and total SCFAs showed a downward trend in patients with ulcerative colitis, whereas acetic acid, butyric acid, and valeric acid showed a downward trend in patients with Crohn’s disease. In addition, subgroup analysis found that the concentration of butyrate in patients with ulcerative colitis changed at different disease stages; it was lower than that in healthy controls in the active stage and higher in the remission stage (88). Future research should focus on the specific role of gut microbiota and their metabolites in the pathogenesis of PD and IBD. Therapeutic strategies should target regulating the composition of gut microbiota and increasing the production of beneficial SCFAs. The researchers should focus on the specific effects of SCFAs on intestinal mucosal permeability and central nervous system inflammation.

## 4 Treatment of PD and IBD

### 4.1 Medicine

Commonly used drugs for the treatment of IBD include non-biological and biological agents. Classical non-biological agents include aminosalicylic acid, thiopurines, and hormones. Biological agents include anti-TNF, interleukin, and other cytokines, and drugs acting on specific inflammation-related pathways (89–91). A 2023 meta-analysis of six studies with data on the use of medications for IBD showed a protective effect of medications for IBD on the onset of PD (RR = 0.88) (92). 5-aminosalicylic acid (5-ASA) and anti-TNF drugs are commonly used for the treatment of IBD. In a cross-sectional study of 144,018 patients with IBD, the risk of PD was lower in those who received anti-TNF drugs than in those who did not (IRR = 0.22; *p* = 0.03) (93). Ríos et al. conducted a study on 20,208,682 patients and found that people under 65 years of age were less likely to receive anti-PD medication while using 5-ASA than those not using 5-ASA (OR = 0.28; *p* = 0.0103) (94). The above findings support the idea that drugs for IBD may indirectly slow the course of PD or reduce the risk of its development by reducing the inflammatory response. The studies on the specific mechanisms underlying the protective effects of IBD drugs on PD, including clinical trials to validate the potential utility of these drugs in patients with PD, may lead to more promising treatment prospects for patients with IBD and PD. The literature suggests that treatment with L-dopa-carbidopa enteric-coated gel (LCIG), which is a commonly used treatment for advanced PD, may indirectly help improve the prevalent GI symptoms in PD patients. Continuous infusion of LCIG is designed to minimize fluctuations in plasma drug concentrations, which not only optimizes motor symptom control but may also help stabilize GI function by providing more stable dopamine stimulation (95). MR Analysis of PD and IBD suggested that the CXCR4 gene is a potential drug target. The gene encodes the chemokine receptor CXCR4, and flavonoids may become potential therapeutic drugs for PD and IBD by inhibiting the CXCR4 protein (96). Therefore, new therapies for PD and IBD can be developed by finding the gene targets of drugs.

### 4.2 Fecal microbiota transplantation and probiotic treatment

FMT and probiotic therapy are two novel approaches for the treatment of gut-related diseases. Both these approaches have been evaluated for the treatment of PD and IBD. These two approaches modulate the gut microbiome and show potential therapeutic value in regulating intestinal inflammation and gut–brain axis interactions. FMT can ameliorate the intestinal microbial imbalance in the mouse model of PD, increase the levels of striatal dopamine and 5-hydroxytryptamine, and play a neuroprotective role by inhibiting neuroinflammation (97). FMT can ameliorate motor symptoms (e.g., tremors and bradykinesia) and non-motor symptoms (such as constipation, anxiety, depression, and sleep disorders) of patients with PD to a certain extent (98, 99). However, FMT is considered a controversial treatment for patients with IBD. Although FMT helps relieve the symptoms of patients with IBD in some small clinical studies, patients become prone to adverse reactions, such as infection and fever (100, 101). In contrast, probiotics are more clinically useful in adjusting intestinal microecology.

According to the definition of the World Health Organization and the Food and Agriculture Organization of the United Nations, probiotics are a group of living microorganisms (102) that can provide health benefits to the host when ingested in appropriate amounts. Probiotics can live and reproduce in the intestinal tract, and have a variety of functions, including maintaining intestinal health, enhancing immunity, promoting nutrient absorption, and alleviating gastrointestinal symptoms. Among them, probiotics perform well in the maintenance of intestinal health. It maintains intestinal health by inhibiting the growth of harmful bacteria, increasing the stability of intestinal mucosal barrier, and promoting intestinal peristalsis (103). Probiotics have recognized antioxidant, anti-inflammatory, and neuroprotective effects, which can regulate central nervous system activity by targeting a variety of cellular and molecular processes, such as oxidative stress, inflammatory and anti-inflammatory pathways, and apoptosis (104). Sun et al. (105) showed that probiotic lactis Probio-M8 synergeted with traditional drug treatment regimens for Parkinson’s disease to enhance the clinical efficacy of PD treatment, while changing the host’s gut microbiome, gut microbial metabolic potential, and serum metabolites. Zhao et al. (106) showed that rotenone-induced PD mouse model damaged the intestinal barrier, leading to the leakage of pathogenic LPS and LBP, which activated the SN and TLR4 signaling pathway in the colon. Fecal microbiota transplantation intervention could protect rotenone-induced PD mouse model by improving the imbalance of intestinal microbiome. Inhibition of the LPS-TLR4 signaling pathway in the gut and brain may play an important role. One study found that long-term use of probiotics produced marked neuroprotective effects on dopaminergic neurons and improved motor deficits in a mouse model of genetic PD (107). The probiotic *E. coli* Nissle1917 is as effective as standard 5-ASA therapy in slowing the progression of ulcerative colitis (108). Daily supplementation of probiotics improved gait, balance function, and motor coordination in a mouse model of PD, and long-term administration of probiotics has a neuroprotective effect on dopamine neurons (109). The results of randomized double-blind controlled trials have indicated that probiotics can help relieve non-motor symptoms, such as constipation, abdominal pain, and bloating, as well as the total Unified Parkinson’s Disease Rating Scale score in patients with PD (110). Although FMT is controversial in the treatment of IBD and may be associated with some risks, it has shown the possibility of alleviating motor and non-motor symptoms in patients with PD. Probiotic therapy, a safer and more clinically accessible treatment, has shown promise in slowing the progression of ulcerative colitis and protecting dopamine neurons in PD models. Future studies are needed to explore the mechanisms of action of FMT and probiotic therapy in the treatment of PD and IBD, especially how they modulate the intestinal microbial community for optimal therapeutic effects. In addition, more clinical trials are necessary to evaluate the safety, efficacy, and long-term effects of these treatments, especially in patients with PD and IBD at different stages and types.

## 5 Conclusion and prospects

The seemingly different diseases, PD and IBD, have many similarities in epidemiological characteristics, pathophysiological mechanisms, and treatment approaches. Therapeutic strategies for IBD, such as anti-inflammatory and immunomodulatory approaches, are potentially protective against PD progression. In addition, modulation of gut microbiota, including interventions through diet, probiotics, or emerging drug targets such as CXCR4, can be used for the treatment of both diseases. The assessment of PD and IBD should adopt a holistic approach, investigating the interrelationship between these two conditions, and deciphering how their interactions influence disease onset and progression. Future research endeavors should focus on elucidating the precise mechanisms underpinning these associations and developing innovative therapeutic approaches to enhance the quality of life for individuals afflicted with these disorders.

## Author contributions

ZW: Conceptualization, Data curation, Formal analysis, Investigation, Validation, Writing – original draft, Writing – review & editing. YJ: Conceptualization, Data curation, Formal analysis, Validation, Writing – original draft, Writing – review & editing. HW: Conceptualization, Formal analysis, Validation, Writing – review & editing. XB: Data curation, Validation, Writing – original draft. WX: Data curation, Validation, Writing – review & editing. JL: Conceptualization, Data curation, Formal analysis, Investigation, Supervision, Validation, Writing – original draft, Writing – review & editing. ZL: Conceptualization, Data curation, Formal analysis, Investigation, Supervision, Validation, Writing – original draft, Writing – review & editing.
